# A Rare Case of Epithelioid Haemangioendothelioma of the Lateral Orbit in a 22-Year-Old Patient

**DOI:** 10.7759/cureus.77084

**Published:** 2025-01-07

**Authors:** Jeffrey Abdullah, Amarpreet Kaur Chohan, Sadiq Mawji, Pradyumna Naredla, Matthew R Idle

**Affiliations:** 1 Oral and Maxillofacial Surgery, Queen Elizabeth Hospital Birmingham, Birmingham, GBR

**Keywords:** diagnosis and management of bony lesions in maxillofacial region, diagnosis and management of mucosal lesions in maxillofacial region, epithelioid haemangioendothelioma, maxillofacial prosthesis, oral and maxillofacial pathology, oral medicine maxillofacial radiology specialist

## Abstract

Epithelioid haemangioendothelioma (EHE) is a rare vascular neoplasm characterised by proliferation of vascular endothelial and pre-endothelial cells. The prevalence is less than one in a million people. It is principally observed in the soft tissues of the extremities but can also occur in the bone, brain, liver, lung and lymph nodes. EHE in the head and neck region is very rare. The most common site of occurrence in the head and neck is the submandibular region. To the best of our knowledge, there are only four cases of EHE reported in the orbit. We herein present the unusual case of a 22-year-old female patient with an EHE of the lateral wall of the right orbit and describe the clinical findings, histopathology, differential diagnoses and treatment. EHEs exhibit the potential for malignancy and recurrence, but metastasis remains rare. Due to its noticeable potential for malignancy and recurrence, complete excision and regular long-term follow-up would be the appropriate treatment protocol.

## Introduction

Epithelioid haemangioendothelioma (EHE) is a rare vascular neoplasm originating from vascular endothelial and pre-endothelial cells [1]. It was first described by Weiss and Enzinger in 1982 as a vascular tumour of the bone and soft tissue with epithelioid appearance, demonstrating features between haemangioma and angiosarcoma [2]. In 2002, the World Health Organization (WHO) described EHE as lesions that fall into the category of locally aggressive tumours with metastatic potential [3].

The prevalence of EHE is less than one in a million people, with a slight predominance in women. The incidence peaks in the fourth to fifth decade [4]. EHEs are usually observed in the extremities, but can also occur in the bone, lung, brain, liver and and lymph nodes [5]. Occurrence of EHEs in the head and neck region is very rare. The most common site of occurrence in the head and neck is the submandibular region, followed by the soft tissues of the neck [6]. There are very few case reports of EHE in the orbit and/or eyelid, with only 11 cases documented: four reported in the orbit and seven in the soft tissues [7-17]. Diagnosis of EHE requires a rigorous investigative process, including scans, biopsies and extensive histopathological testing. The differential diagnoses for EHE can include epithelioid haemangioma, epithelioid sarcoma, epithelioid angiosarcoma, and metastatic carcinoma [15]. 

In this paper, we report the case of a 22-year-old female with an EHE of the right lateral wall of the orbit.

## Case presentation

A 22-year-old Caucasian female initially presented to her general practitioner (GP) with a painful swelling at the right lateral canthus that started in August 2023, whilst the patient was in the first trimester of pregnancy. The patient reported a painful swelling on the lateral aspect of her right eye, with associated double vision and excess tears. She had a medical history of polycystic ovary syndrome, did not take any regular medications and had no known drug allergies. She had never smoked and did not drink alcohol.

The GP treated the patient with antibiotics for a suspected orbital cellulitis. However, the swelling gradually increased in size and she was experiencing right-sided headaches more frequently. The patient independently sought an ophthalmology opinion where a diagnosis of meningioma was suspected, and she was subsequently referred for neurosurgical input. 

**Figure 1:**
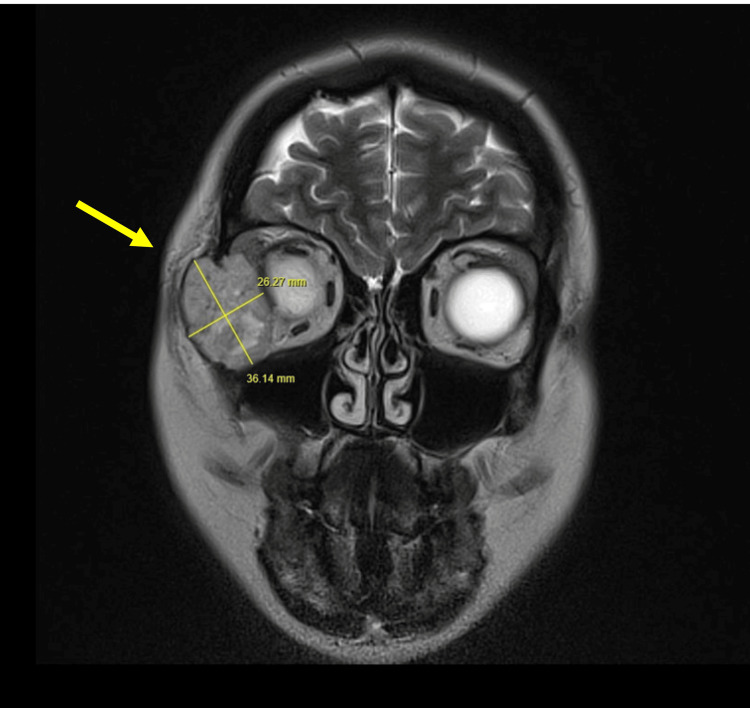
Coronal view of the MRI head with a well-defined mass (indicated by the arrow) at the right lateral orbit

Under the neurosurgical team the patient had MRI and CT of the head in June 2024. The MRI demonstrated a well-defined mass along the lateral wall of the right orbit with displacement of the surrounding fat, tissue planes and right globe (Figure 1).

The findings at the time were compatible with meningioma. A subsequent CT head showed an expansile bony lesion involving the lateral wall of the right orbit measuring 40 x 28 x 32 mm (40 mm in craniocaudal dimension, 28 mm in maximum transverse dimension, 32 mm in maximum anterior-posterior dimension). The lesion contained calcific septation, with encroachment and displacement of the right lateral rectus medially, and mild compression of the right globe. There is a degree of radiological exophthalmos and the surrounding soft tissue appeared normal. 

**Figure 2:**
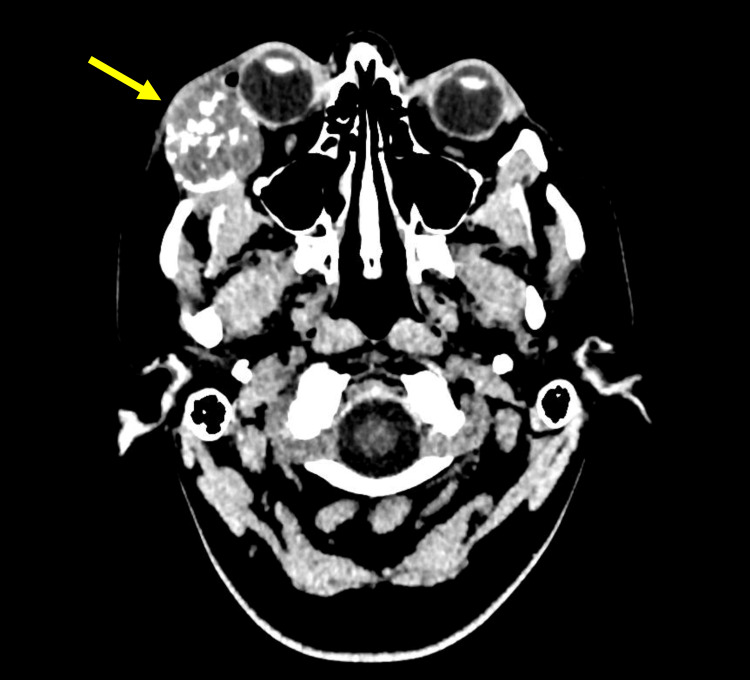
Axial view of the CT head demonstrating the bony lesion (indicated by the arrow) at the right lateral orbit

In conjunction with the MRI, this was reported as suggestive of Ewing’s sarcoma of the right lateral orbit (Figure 2).

The patient was referred to the sarcoma multidisciplinary team (MDT) and our oral and maxillofacial surgery (OMFS) service. She attended our department in September 2024 reporting the same history of presenting complaint as to her GP. Due to the lapsed time, this was now a one-year history and the patient was four months postpartum.

**Figure 3:**
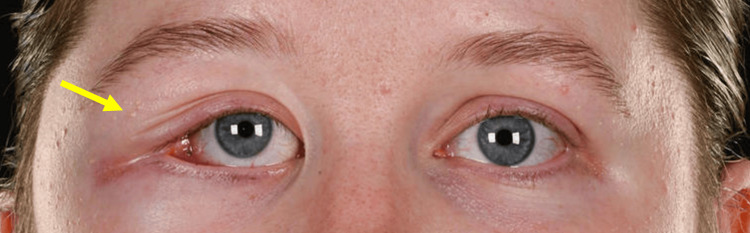
Clinical photograph demonstrating hyperglobus of the right eye and displacement of the right lateral canthus (indicated by the arrow)

**Figure 4:**
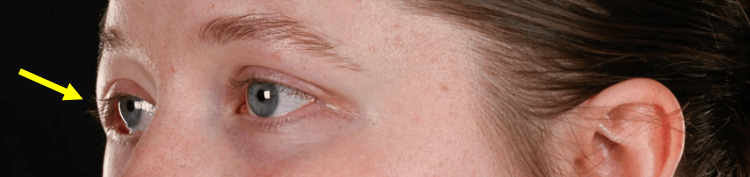
Left facial view demonstrating displacement of the right lateral canthus (as indicated by the arrow)

**Figure 5:**
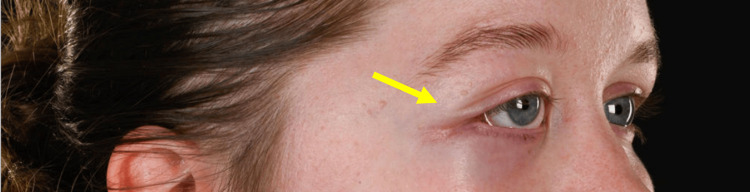
Right-sided view of the right lateral canthus (as indicated by the arrow)

On examination by the maxillofacial team, there was no cervical lymphadenopathy noted. There was a noticeable firm swelling at the lateral aspect of the right eye. There was mild proptosis and hyperglobus of the right eye and some displacement of the right lateral canthus (Figures 3-5). She had a full range of eye movement, with diplopia reported on extreme lateral gaze. Visual acuity was otherwise unremarkable. Furthermore, the swelling was palpable intraorally in the buccal sulcus of the upper right quadrant. 

An urgent incisional biopsy of the lesion was performed via an intraoral approach. Microscopically, the sections showed bony tissue infiltrated by solid sheets of atypical malignant cells with mixed spindle cell and epithelioid morphology. These cells were enlarged with pleomorphic irregular nuclei, large central prominent nucleoli and amphophilic cytoplasm. Many cells showed intranuclear inclusions and cytoplasmic vacuoles with possible cytoplasmic red blood cells. There were also transformed malignant cells with bizarre multinucleated cells noted. There was no evidence of necrosis, and mitosis was rarely seen, meaning there did not appear to be a high-grade malignancy. Overall, these features were consistent with a vascular neoplasm, with EHE and epithelioid angiosarcoma regarded as possible diagnoses. 

To confirm the diagnosis of EHE, a next-generation sequencing (NGS) molecular test was performed. Total RNA was extracted from the tissue sample with an estimated lesional content of 80%. The targeted NGS assay was used to detect and identify gene fusions involving the 63 genes associated with soft tissue cancers. CAMTA1 gene fusion was detected, which is a recurrent finding in EHE.

**Figure 6:**
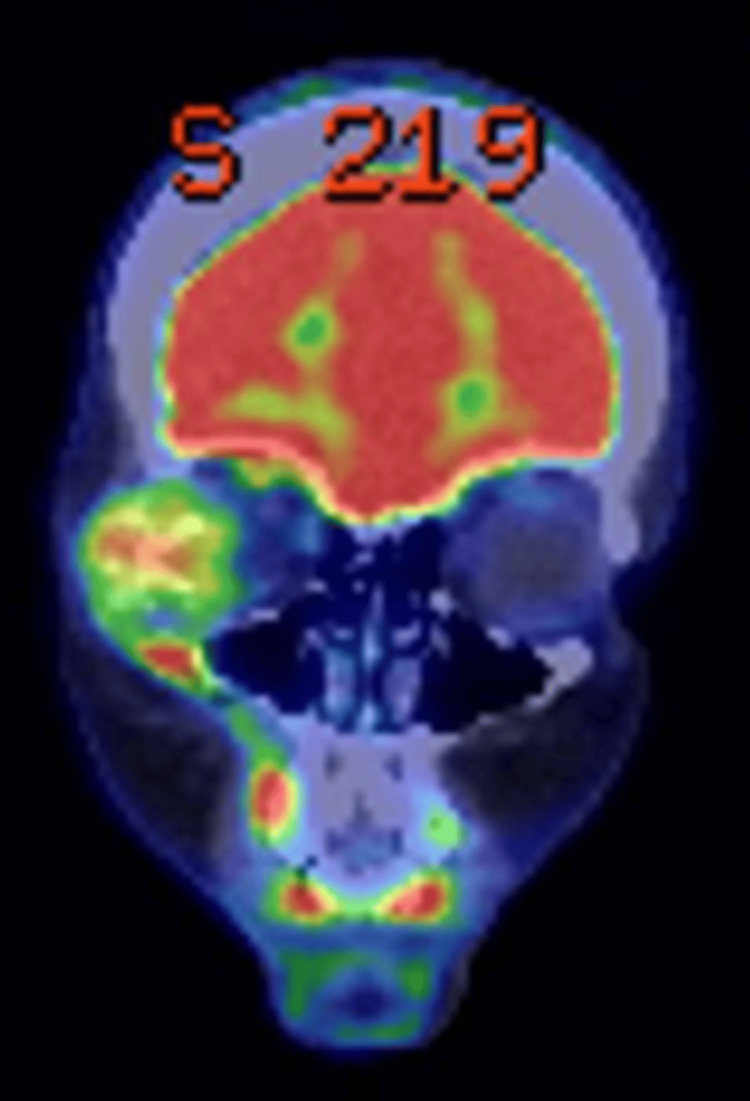
PET-CT demonstrating the metabolically active expansile mass at the right orbit PET: Positron emission tomography

A whole-body positron emission tomography (PET)-CT was requested, and it demonstrated a metabolically active expansile mass in the right orbital wall and zygomatic process with no evidence of metastatic disease (Figure 6).

Following discussions at the sarcoma MDT, surgical resection of the EHE at the right lateral orbit was recommended, with acknowledgement of a close surgical margin where the lesion contacts the right orbital contents. 3D planning allowed for a custom-made orbit and zygomatic implant to be constructed.

The surgical resection took place two weeks after the MDT. A transconjunctival incision with lateral canthotomy was used for access. Osteotomies of the zygomaticofrontal suture, infraorbital rim and zygomatic arch were performed. The fracture was propagated through the lateral orbital rim onto the sphenoid bone, and the tumour was removed as a whole. The titanium prosthesis was inserted and secured with screws. Primary closure was achieved. The forced duction test was used to evaluate ocular movements and demonstrated no concerns. There were no immediate surgical complications and the patient was discharged within 48 hours.

The pathology results reported that the resection margins were clear from tumour. The case was rediscussed at MDT and the recommendation was to repeat an MRI scan in three months, with clinical reviews in the interim.

## Discussion

EHE of the orbit is incredibly rare. To the best of our knowledge, there have been only four cases documented previously. 

The clinical presentation of orbital EHE can vary, as it is dependent on the site of the tumour and the level of compression it exerts on the surrounding tissues [15]. In this instance, as the firm mass occurred at the lateral wall of the right orbit, there was mild ocular proptosis and hyperglobus with diplopia on extreme lateral gaze. The patient’s visual acuity was preserved. 

**Figure 7:**
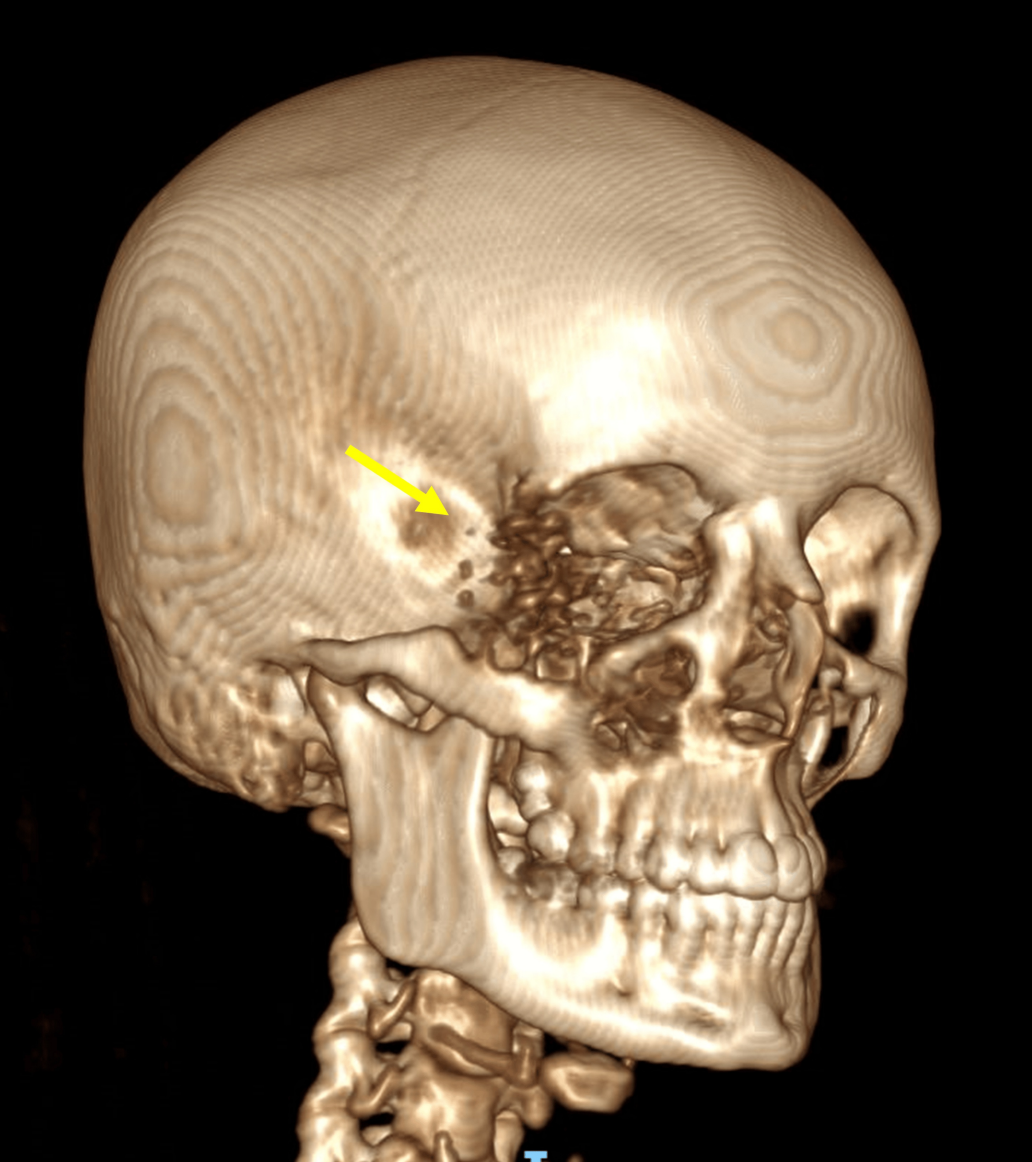
3D right-sided view of the CT head demonstrating destruction of the right orbit and zygoma (indicated by the arrow)

**Figure 8:**
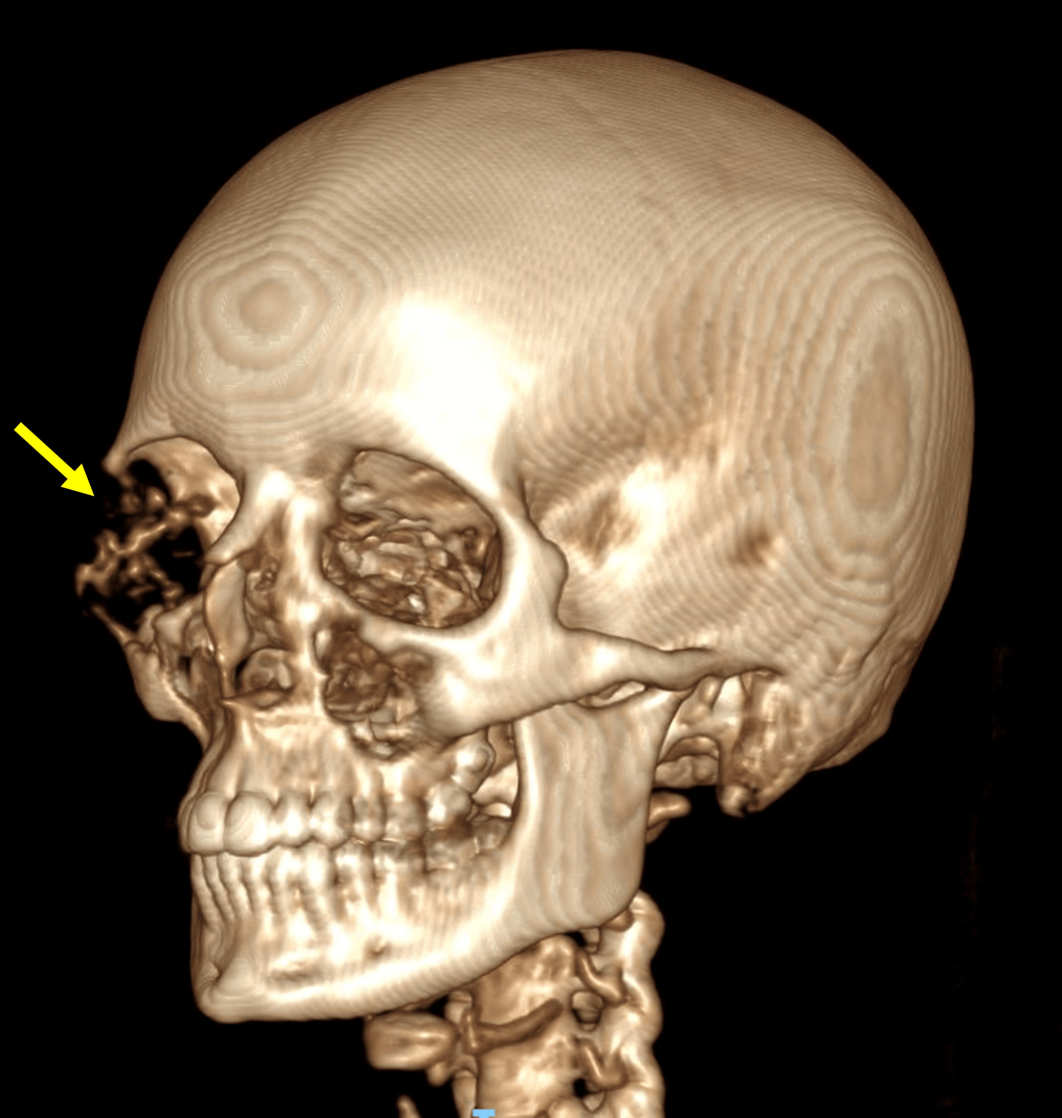
3D left-sided view of the CT head demonstrating destruction of the right orbit and zygoma (indicated by the arrow)

The diagnosis of orbital EHE can be challenging. This is due to its rarity and the fact that there are very few literature reports and case series available. In this case, the patient was misdiagnosed with orbital cellulitis, meningioma and Ewing’s sarcoma, highlighting EHE’s diagnostic difficulty. For orbital tumours, the main imaging techniques should consist of CT and MRI. These allow for an assessment of the vascular nature of the tumour and the effect of the mass on the adjacent structures [15]. A review of the available literature showed that bony EHEs cause obvious destruction of the bone, whereas soft tissue EHEs tend to grow without invading the neighbouring tissues [15]. This patient’s CT clearly demonstrated an expansile lesion with associated destruction of the right orbit and zygoma (Figures 7,8). 

Histopathology and immunohistochemistry are key to diagnosis. Histopathological examination of EHE should reveal the presence of epithelioid cells. These are round, oval or polygonal shaped cells that form the primitive blood vessels of the tumour. Another characteristic feature of EHE is vacuolation of the cytoplasm, which can be empty in appearance or contain red blood cells [18]. These were all evident in the present case.

Although there were many suggested diagnoses for this patient, the histopathological report regarded EHE and epithelioid angiosarcoma as differential diagnoses. However, epithelioid angiosarcoma is often associated with irregular vascular lumens, necrosis and high mitotic figures [2]. These features were not identified in our case. Moreover, with the aid of molecular genetic studies, these can be utilised to detect chromosomal translocations specific to EHE. In EHE, the fusion gene produced is usually the CAMTA1 gene, while in a small proportion of cases, the TFE3 gene is observed [19]. In our case, immunohistochemistry with CAMTA1 and TFE3 markers were used to detect these specific gene translocations. With the CAMTA1 fusion gene identified, a diagnosis of EHE was affirmed. 

**Figure 9:**
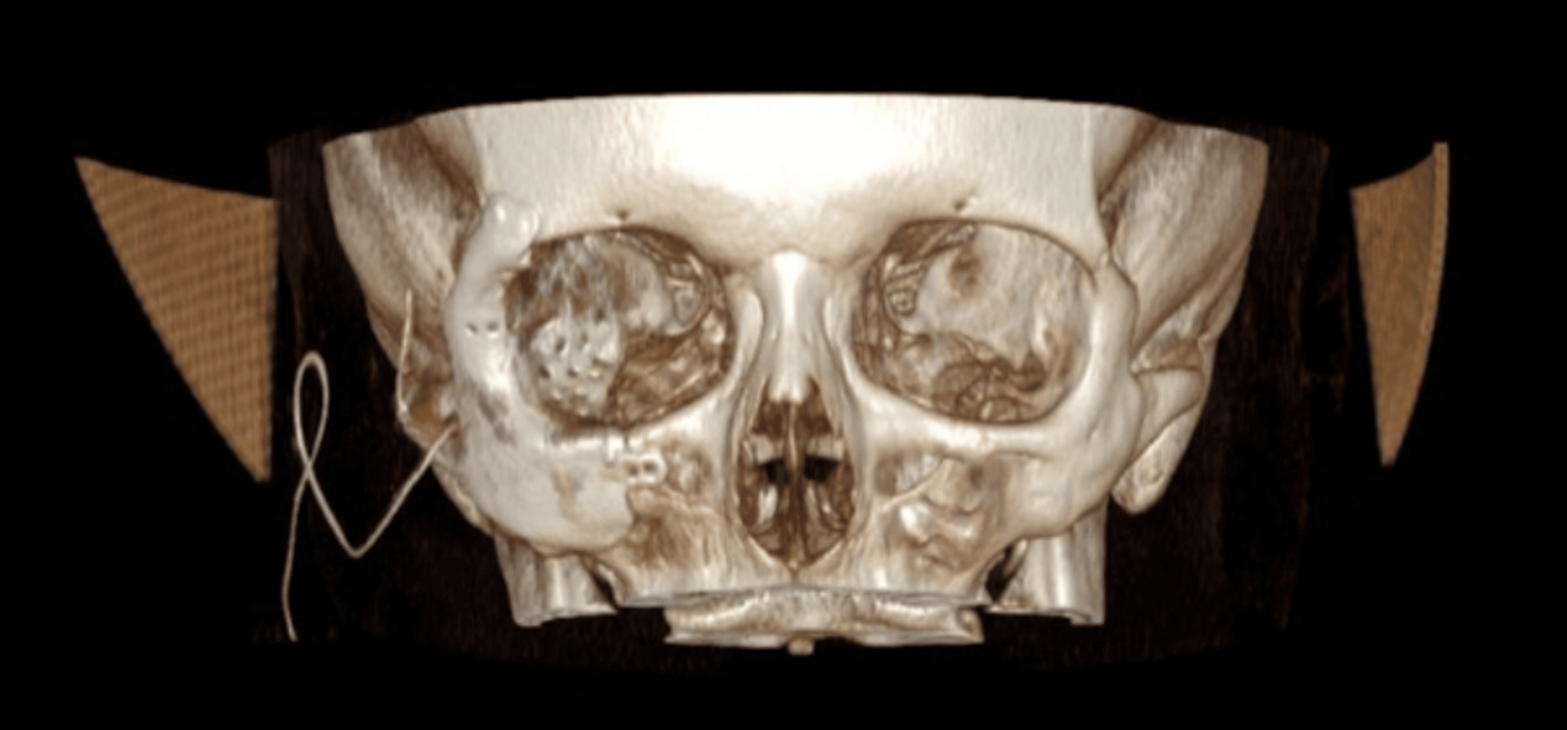
3D frontal view of CT head with right orbito-zygomatic implant

**Figure 10:**
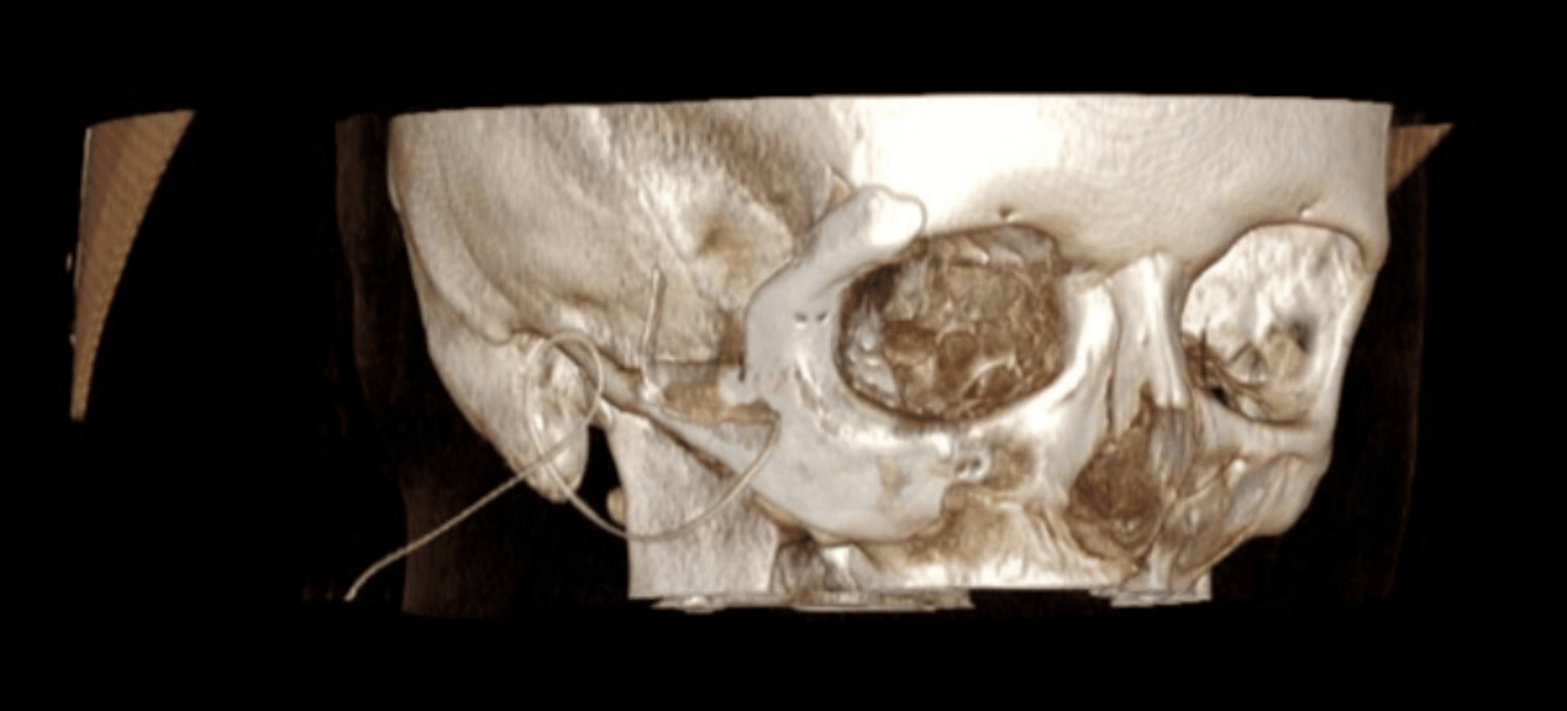
3D right-sided view of CT head with right orbito-zygomatic implant

**Figure 11:**
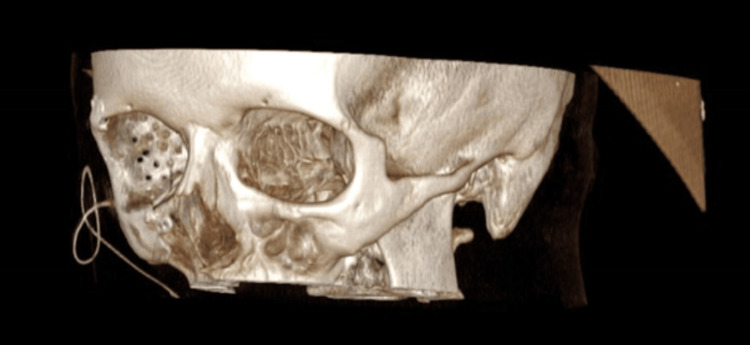
3D left-sided view of CT head with right orbito-zygomatic implant

At present, the primary treatment modality for localised EHE is complete surgical resection. An increased risk of local recurrence is usually related to incomplete excision. However, factors that hinder complete excision can depend on the site of the tumour, its extension and its bleeding potential [15]. Following discussions at the sarcoma MDT, our patient underwent complete excision of the vascular tumour with placement of a right orbito-zygomatic implant (Figures 9-11). 

**Figure 12:**
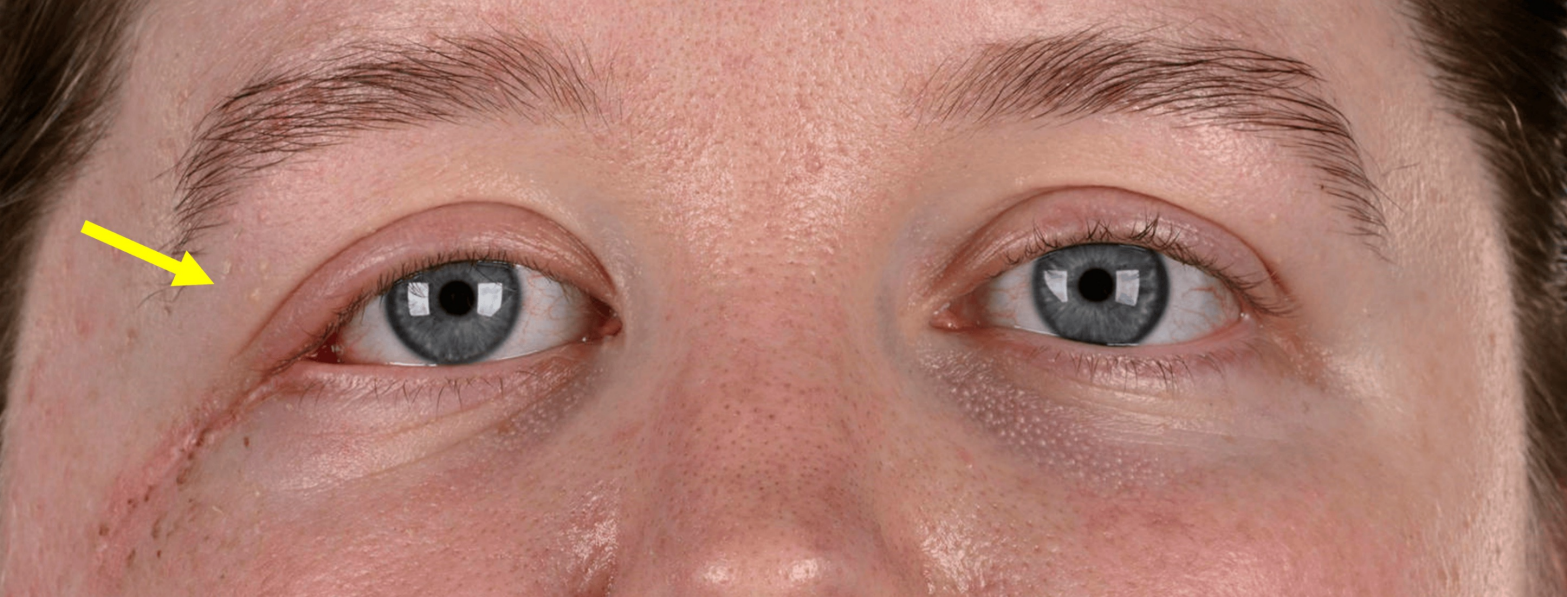
Clinical photograph taken two weeks following excision of the EHE from the right orbit (indicated by the arrow) EHE: Epithelioid haemangioendothelioma

**Figure 13:**
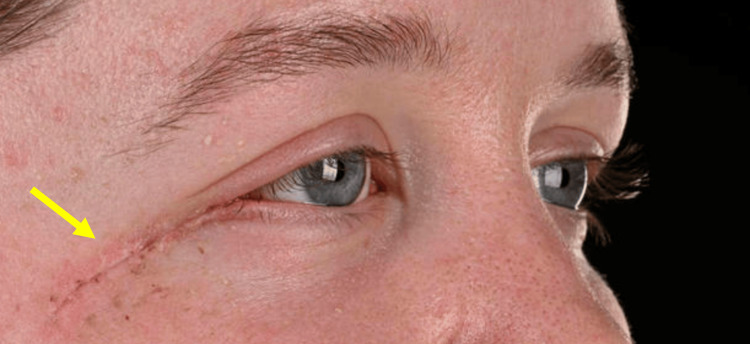
Right-sided view demonstrating good healing of the skin two weeks following excision of the EHE from the right orbit (indicated by the arrow) EHE: Epithelioid haemangioendothelioma

Clinical photographs were taken two weeks post-operatively (Figures 12, 13).

## Conclusions

In conclusion, diagnostic accuracy is critical to allow for accurate treatment of EHE, and surgical resection remains the mainstay of treatment. There are no reported cases of metastatic orbital EHE thus far. The risk of distant metastases of orbital EHEs is therefore difficult to predict. Nevertheless, due to EHE’s noticeable potential for malignancy and recurrence elsewhere in the body, regular long-term follow-up of orbital EHE with MRI should be recommended. 
